# Automatic UAV-based detection of *Cynodon dactylon* for site-specific vineyard management

**DOI:** 10.1371/journal.pone.0218132

**Published:** 2019-06-11

**Authors:** Francisco Manuel Jiménez-Brenes, Francisca López-Granados, Jorge Torres-Sánchez, José Manuel Peña, Pilar Ramírez, Isabel Luisa Castillejo-González, Ana Isabel de Castro

**Affiliations:** 1 Crop Protection Department, Institute for Sustainable Agriculture (IAS), Spanish National Research Council (CSIC), Córdoba, Spain; 2 Plant Protection Department, Institute of Agricultural Sciences (ICA), Spanish National Research Council (CSIC), Madrid, Spain; 3 Crop Production Department, Andalusian Institute of Agricultural and Fisheries Research and Training (IFAPA), Cabra, Córdoba, Spain; 4 Department of Graphic Engineering and Geomatics, University of Córdoba, Córdoba, Spain; California State University Fresno, UNITED STATES

## Abstract

The perennial and stoloniferous weed, *Cynodon dactylon* (L.) Pers. (bermudagrass), is a serious problem in vineyards. The spectral similarity between bermudagrass and grapevines makes discrimination of the two species, based solely on spectral information from multi-band imaging sensor, unfeasible. However, that challenge can be overcome by use of object-based image analysis (OBIA) and ultra-high spatial resolution Unmanned Aerial Vehicle (UAV) images. This research aimed to automatically, accurately, and rapidly map bermudagrass and design maps for its management. Aerial images of two vineyards were captured using two multispectral cameras (RGB and RGNIR) attached to a UAV. First, spectral analysis was performed to select the optimum vegetation index (VI) for bermudagrass discrimination from bare soil. Then, the VI-based OBIA algorithm developed for each camera automatically mapped the grapevines, bermudagrass, and bare soil (accuracies greater than 97.7%). Finally, site-specific management maps were generated. Combining UAV imagery and a robust OBIA algorithm allowed the automatic mapping of bermudagrass. Analysis of the classified area made it possible to quantify grapevine growth and revealed expansion of bermudagrass infested areas. The generated bermudagrass maps could help farmers improve weed control through a well-programmed strategy. Therefore, the developed OBIA algorithm offers valuable geo-spatial information for designing site-specific bermudagrass management strategies leading farmers to potentially reduce herbicide use as well as optimize fuel, field operating time, and costs.

## Introduction

Vineyard yield and grape quality are variable as a consequence of intrinsic factors related to the crop and the field [1]. However, most vineyards have been managed as homogenous parcels of land due to the absence of methods that accurately analyze variability [2]. Therefore, analysis of the influence and spatial distribution of variability will allow grape growers to manage vineyards more efficiently for production and grape quality [3]. This approach is the agronomic basis of precision viticulture (PV), which assesses within-field spatial variability (e.g., soil characteristics, weed patches, fungi infection, insect pest attack, grape quality or maturation, production, balance between vegetative growth, and reproductive growth, among others) [4]. Implementation of PV, for either targeted management of inputs and/or selective harvesting at vintage, begins with monitoring vineyard performance and associated attributes, followed by interpretation and evaluation of the collected data [5]. PV is mainly focused on optimizing crop production and profitability by reducing production inputs;, therefore, its main objective is to diminish the potential damage to the environment and unnecessary costs due to over-application of inputs. Besides these economic and environmental benefits, PV practices comply with the European Policy to regulate a sustainable and rational use of agricultural products and pesticides at a farm level to lead current climatic, socio-economic, and environmental changes while ensuring feasibility and profitability [6].

Remote sensing has been widely used to characterize vineyards and their associated attributes to be used in site-specific management. For example, [7,8] explored satellite images to predict wine yield and map vineyard leaf area, respectively; [9] used images taken by piloted aircrafts to estimate the grapevine canopy density and identify the grapevine rows. Currently, Unmanned Aerial Vehicles (UAVs) stand out among the other remote sensing platforms because they can fly at low altitudes, capture images with ultra-high spatial resolution (millimetric accuracy) [3,10,11], and, on demand in critical moments, which are not feasible with airborne or satellite platforms. Therefore, the use of UAVs has been proven to be a crucial remote sensing tool to address PV objectives [12–14].

Weeds are known to be a major problem in agriculture, leading to a 32% worldwide reduction in crop yields [15]. Recently, *Cynodon dactylon* (L.) Pers. (bermudagrass) has been reported to infest vineyards [16,17], causing competition for nutrients and water, especially in summer when irrigation is needed [18]. This perennial summer grass is widely adapted to a range of climates and soils, propagates mainly vegetatively through stolons and rhizome fragmentation, and is considered a serious problem in cultivated crops worldwide. In addition, weed management strategies in vineyards such as tillage, herbicides, or cover crops have strong implications for wine quality [19–21].

The spectral similarity between bermudagrass and grapevines in summer just, when competition for water is maximum and weeds must be controlled, makes discrimination using pixel-based image analysis almost unfeasible, as this approach focuses solely on spectral information [22]. Alternatively, the use of UAV-based Digital Surface Models (DSMs) has been shown to be an efficient alternative to isolate and classify woody crop plants [3,23,24]. Nevertheless, computing the large amount of data embedded in UAV images and DSMs requires the implementation of robust and automatic image analysis procedures. In this sense, object-based image analysis techniques (OBIA) have reached high levels of automation and adaptability to ultra-high spatial resolution images, typical of UAV images [25,26]. Compared to pixel-based methods, the application of object-based approach offers the possibility of evaluating spectral and textural, contextual, and hierarchical features [27], addressing challenging spectral similarity scenarios related to the design of site-specific weed management [25]. However, to the best of our knowledge, the UAV-based DSM and OBIA combination has not yet been applied to map bermudagrass in vineyards.

Therefore, the goal of this research was automatic, accurate, and rapid mapping of bermudagrass and designing management maps using UAV-imagery and OBIA techniques. The specific objectives included: (1) selection of the optimum spectral vegetation indices that best discriminated bermudagrass from bare soil as affected by sensors separately attached to the UAV (spectral analysis); (2) development of an automatic and robust OBIA algorithm for each camera, using those selected vegetation indices, for classifying bermudagrass, bare soil, and grapevines and evaluating the accuracy of the procedure (image analysis); and (3) design of site-specific management maps according to weed infestation level. It is important to highlight that the full protocol established in this paper is composed of a novel OBIA algorithm that does not require user intervention.

## Materials and methods

### Study sites description and UAV flights

This research was conducted in two experimental drip-irrigated organic vineyards, fields A and B, located in Cabra (Córdoba, Southern Spain). Each site was approximately 0.5 hectares. Both vineyards were planted with cv. Pedro Ximénez in 2013 with rows oriented east–west and trained as a vertical shoot positioned bilateral cordon. Plant spacing was 2.5 m (inter-rows) x 1.3 m (intra-row). Inter-row spaces were uniformly managed by biannual tillage and manual mowing using a brush cutter, which effectively controlled all weed species except bermudagrass, resulting in clean inter-row spacing without cover green and only with the presence of bermudagrass patches.

**Fig 1:**
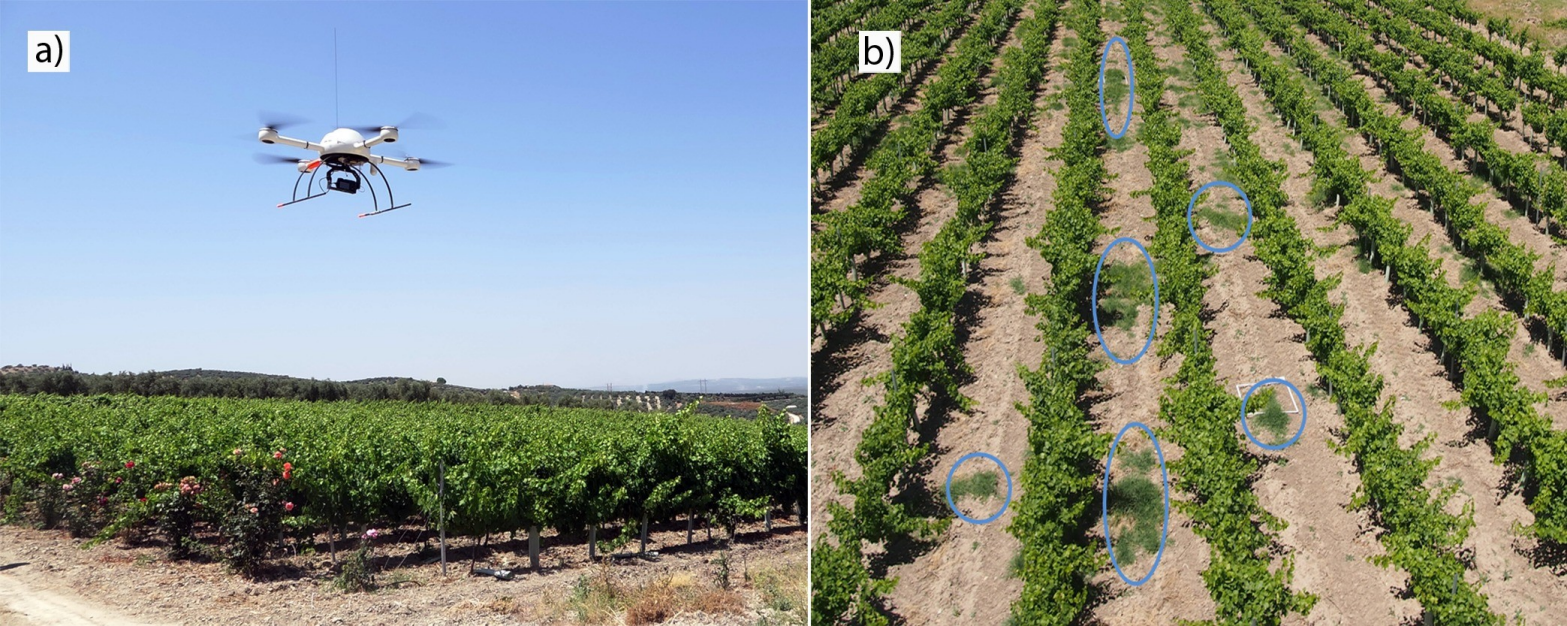
a) Quadcopter microdrone MD4-1000 with the Red-Green-Near Infrared (RGNIR) camera attached, flying over one of the vineyards and b) detail of an RGB-image taken by the UAV from field A-2017. The circles in blue color represent bermudagrass patches growing in the inter-rows.

A quadcopter model MD4-1000 (microdrones GmbH, Siegen, Germany) with vertical take-off and landing (Fig 1A) was used as the platform for image acquisition. This model with four brushless motors was battery-powered and could either be manually operated by radio control or autonomously with the aid of its Global Position System (GPS) receiver and its waypoint navigation system. The imagery were acquired with two still point-and-shoot cameras that were separately mounted in the UAV: (1) a visible-light (RGB: Red (R), Green (G) and Blue (B)) camera, model Olympus PEN E-PM1 (Olympus Corporation, Tokyo, Japan) with a sensor size of 17.3 x 13.0 mm and 12.2 megapixels (4,032 x 3,024 pixels); and (2) a modified (RGNIR: Red (R), Green (G) and NIR) camera, model SONY ILCE-6000 (Sony Corporation, Tokyo, Japan) composed of a 23.5 × 15.6 mm APS-C CMOS sensor capable of acquiring 24 megapixels (6000 × 4000 pixels). The RGNIR camera was modified to capture information in both the NIR and visible light (green and red) by adding a 49-mm filter ring to the front nose of the lens, manufactured by Mosaicmill (Mosaicmill Oy, Vantaa, Finlandia), where a focus calibration process was carried out.

The flight missions were conducted in mid-June 2016 (field A) and 2017 (fields A and B), when bermudagrass was at the vegetative growth stage, showing the typical green color of this phenological stage (Fig 1B), and, therefore, had a spectral response very similar to that of the grapevines. During each flight, the UAV route was configured to fly at 30 meters altitude with a forward lap of at least 90%. In addition, a side lap of 60% was programmed. The flights were carried out at noon, to take advantage of the sun’s position and thus minimize shadows on acquired images. All flight operations fulfilled the list of requirements established by the Spanish National Agency of Aerial Security including pilot license, safety regulations, and limited flight distance [28].

### Geomatic products generation

**Fig 2:**
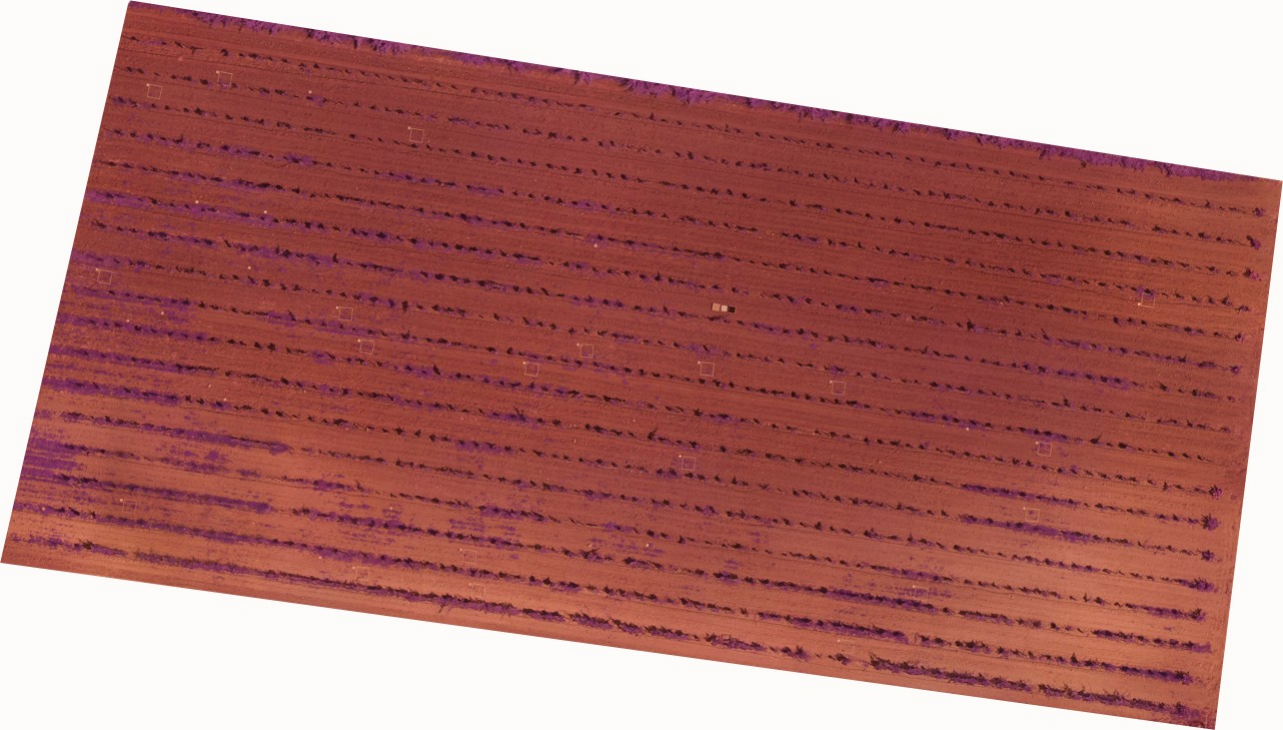
RGNIR orthomosaic corresponding to field A-2016.

The images acquired from each camera were processed using PhotoScan Professional software, version 1.2.4 build 2399 (Agisoft LLC, St. Petersburg, Russia) to generate three geomatic products: (1) a three-dimensional (3D) point cloud, by applying the Structure-from-Motion (SfM) technique; (2) a digital surface model (DSM) created from the 3D point cloud that provides height information; and (3) an orthomosaic (Fig 2), where every pixel contained RGB or RGNIR information depending on the camera used as well as the spatial information.

The mosaicking process was fully automatic, except for the manual localization of six ground control points (GCPs), with four placed in the corners and two in the center of each field to georeference the geomatic products. These GCPs coordinates were measured using two GNSS receivers: one was a reference station from the GNSS RAP network from the Institute for Statistics and Cartography of Andalusia (Spain), and the other was a GPS with a centimeter accuracy (model Trimble R4, Trimble company, Sunnyvale, California, United States) as a rover receiver. First, the software matched the camera position and common points for each image, which facilitated the refinement of the camera calibration parameters. Once the images were aligned, the 3D point cloud was generated by applying SfM technique to the images, which was used as the basis to generate the DSM. The DSM represents the irregular geometry of the ground and the objects on it by means of a 3D polygon mesh. Next, the individual images were projected over the DSM, and the orthomosaicked image was generated. Finally, the DSM was joined to the orthomosaic as a TIFF file consisting of a 4-band multi-layer file (Red, Green, Blue and DSM, for the visible-light camera; and Red, Green, NIR, and DSM, for the modified one). A further description about the PhotoScan function is given in [29].

The geomatic products had different spatial resolutions according to the technical characteristics of each sensor. For example, in 2016: (1) 0.86 and 1.72 cm/pixel for the orthomosaic and DSM generated from the RGB camera; and (2) 0.54 and 1.07 cm/pixel for the RGNIR camera, which was almost half of the values obtained with the RGB camera. The methodology to build these accurate geomatic products has been validated in previous studies [24].

### Ground truth data

**Fig 3:**
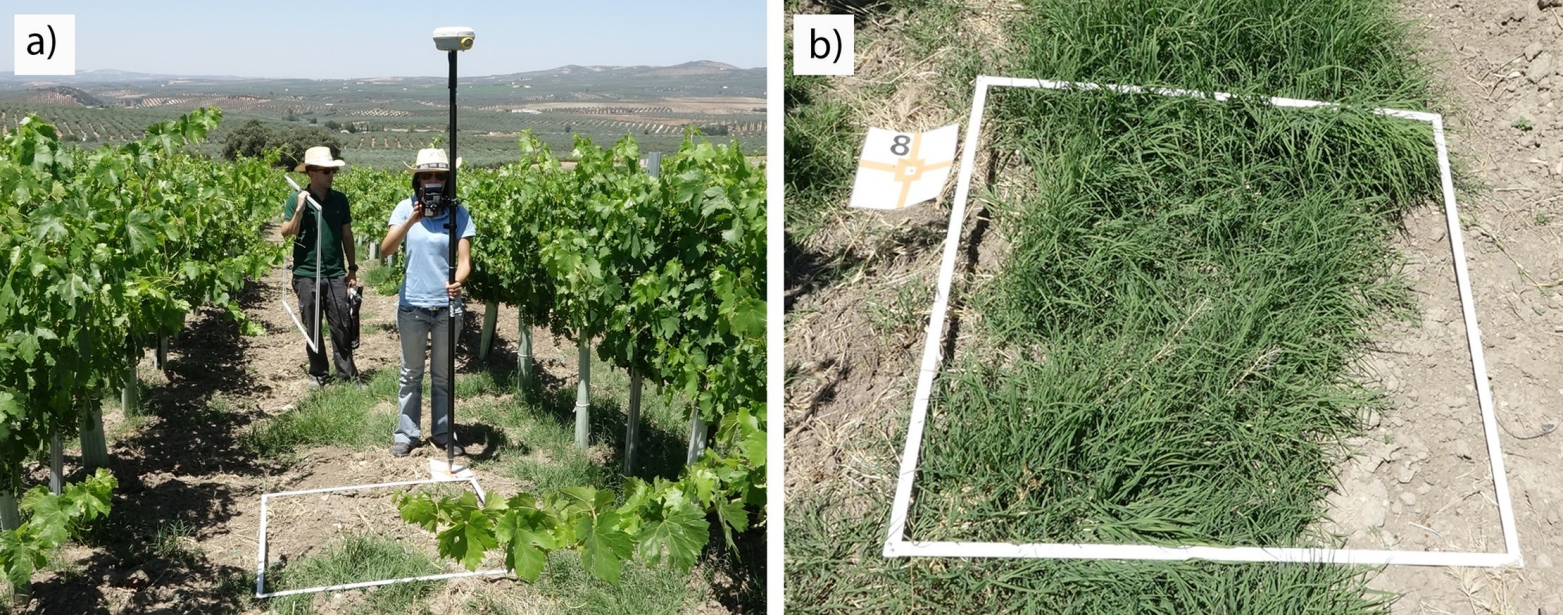
a) Placing and georeferencing the frames in field A-2017 and b) detail of a frame covering bermudagrass and bare soil classes. The individuals in this manuscript have given written informed consent (as outlined in PLOS consent form) to publish these case details.

A set of 18 1 x 1 m georeferenced sampling frames was placed in every field to represent the current weed infestation in the vineyard, ensuring that the entire field had an equal chance of being sampled without operator bias [30]. The frames were set covering bare soil and bermudagrass patches, and georeferenced as described for the GCPs (Fig 3).

**Fig 4:**
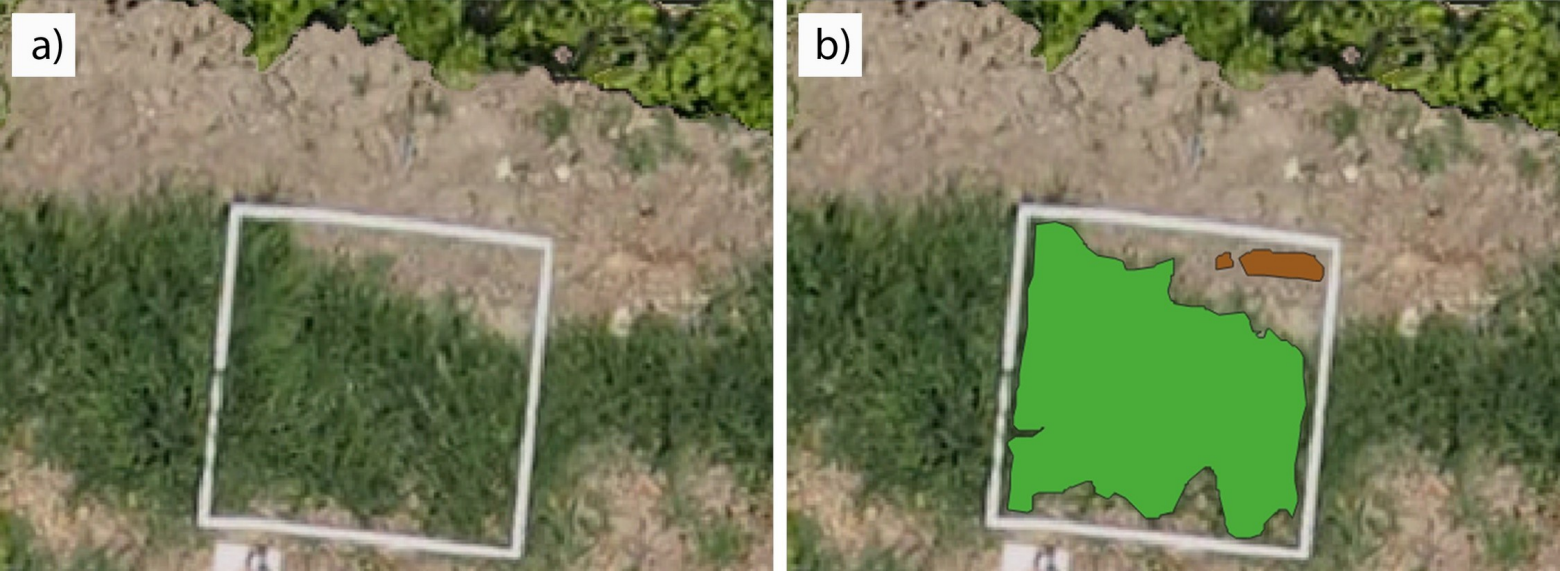
Detail of RGB-orthomosaic of field A-2017 showing: a) sampling frames covering bermudagrass and bare soil and b) manual classification of bermudagrass (green color) and bare soil (brown color) classes that made up the ground truth data.

The high resolution of the orthomosaic (Fig 4A) made it possible to visually identify the bermudagrass patches in every sampling frame and conduct a manual classification of weed infestation and bare-soil (Fig 4B) using ENVI software (Exelis Visual Information, Solutions, Boulder, Colorado, United States), which resulted in the ground truth (GT) data for the procedure. 25% of the GT full dataset corresponding to field A-2016 as well as 25% the GT full dataset of field A-2017 were used for the spectral analysis, whereas the remaining 75% of every field-year were employed for the validation of the image analysis (OBIA algorithm) of each orthomosaic. Additionally, field B-2017 was selected to generalize the procedure, using the GT full dataset only for validation purposes of the classification of bermudagrass infestation map.

### Spectral analysis: Optimum vegetation index

In order to spectrally separate bare soil and bermudagrass, the following analysis was performed. As explained above, 25% of the GT full dataset from both the A-2016 and A-2017 fields was used in the spectral analysis to select the optimal vegetation index (VI) that best discriminated bermudagrass and bare soil for each camera (visible and modified). The mean spectral reflectance calculated for the three spectral bands of each camera (RGB and RGNIR) for each class (weed and bare soil) were used to calculate 14 and 18 vegetation indices and band ratios, respectively (Table 1). The VIs in this study are related to vegetation conditions and plant structure and are widely used in agricultural studies [31,32].

**Table 1:** Spectral vegetation indices and their equations used for both cameras.

Vegetation index	Equation	Camera^a^
R/B index [34]	$\frac{R}{B}$	1
R/G index *(This study)*	$\frac{R}{G}$	1, 2
Normalized Red Green difference index [35]	$NRGDI=\frac{G-R}{G+R}$	1, 2
Normalized pigment chlorophyll index [36]	$NPCI=\frac{R-B}{R+B}$	1
Visible atmospherically resistant index [37]	$VARI=\frac{G-R}{G+R-B}$	1
Woebbecke index [38]	$WI=\frac{G-B}{R-G}$	1
Excess Blue [39]	*ExB* = 1.4 *B*−*G*	1
Excess Green [40]	*ExG* = 2 *G*−*R*−*B*	1
Excess Red [41]	*ExR* = 1.4 *R*−*G*	1, 2
Excess Green-Red [42]	*ExGR* = *ExG*−*ExR*	1
Color index of vegetation [43]	*CIVE* = 0.441 *R*−0.811 *G*+0.385 *B*+18.78745	1
Vegetative index [44]	$VEG=\frac{G}{\left(R^{0.667})\times (B^{1-0.667}\right)}$	1
Indices combination1 [39]	*COMB*1 = 0.25 *ExG*+0.3 *ExGR*+0.33 *CIVE*+0.12 *VEG*	1
Indices combination2 [45]	*COMB*2 = 0.36 *ExG*+0.47 *CIVE*+0.17 *VEG*	1
Chlorophyll index green [46]	$CI=\frac{NIR}{G}-1$	2
Difference vegetation index [47]	*DVI* = *NIR*−*R*	2
Vegetation index faster [48]	$VIF=\frac{NIR}{NIR+R}$	2
Green normalized difference vegetation index [49]	$GNDVI=\frac{NIR-G}{NIR+G}$	2
Ratio vegetation index [50]	$RVI=\frac{R}{NIR}$	2
Modified normalized difference vegetation index [51]	$MRVI=\frac{RVI-1}{RVI+1}$	2
Modified simple ratio [52]	$MSR=\frac{\frac{NIR}{R}-1}{\sqrt{\frac{NIR}{R}+1}}$	2
Modified soil-adjusted vegetation Index [53]	$MSAVI=\frac{2NIR+1-\sqrt{\left({2NIR+1)}^{2}-8\times (NIR-R\right)}}{2}$	2
NIR–G index [54]	*NIR*−*G*	2
NIR/G index [54]	$\frac{NIR}{G}$	2
Non-linear vegetation index [55]	$NLI=\frac{{NIR}^{2}-R}{{NIR}^{2}+R}$	2
Normalized difference vegetation Index [56]	$NDVI=\frac{NIR-R}{NIR+R}$	2
Optimization soil-adjusted vegetation index [57]	$OSAVI=\frac{NIR-R}{NIR+R+0.16}$	2
Transformed vegetation index 1 [58]	$TVI1=\frac{NDVI+0.5}{ABS(NDVI+0.5)}\times \sqrt{ABS(NDVI+0.5})$	2
Transformed vegetation index 2 [59]	*TVI*2 = 0.5×(120×(*NIR*−*G*)−200×(*R*−*G*))	2

^a^1: RGB; 2: RGNIR

The VIs were analyzed by performing a one-way analysis of variance (ANOVA) followed by Tukey´s Honest Significant Difference test (P<0.05) and finally, applying the M-statistic (Eq 1) [33] to quantify the histogram separation of vegetation indices. The M-statistic value expresses the difference in the means of the class 1 and class 2 histograms normalized by the sum of their standard deviations (σ). According to [33], the same difference in means can give different measures of separability depending on the spread of the histograms, i.e., narrow histograms (smaller σ) will cause less overlap and more separability than wider histograms for the same difference in means.

$$M=\frac{{Mean}_{class1}-{Mean}_{class2}}{\sigma_{class1}+\sigma_{class2}}$$ (1)

Statistical analysis was conducted using the software JMP (JMP 10, SAS Institute Inc., Campus Drive, Cary, NC, USA 27513). The selected VI for each camera was subsequently implemented in the OBIA algorithm for bermudagrass, bare soil, and grapevine classification.

### Image analysis: Bermudagrass mapping

#### OBIA algorithm

**Fig 5:**
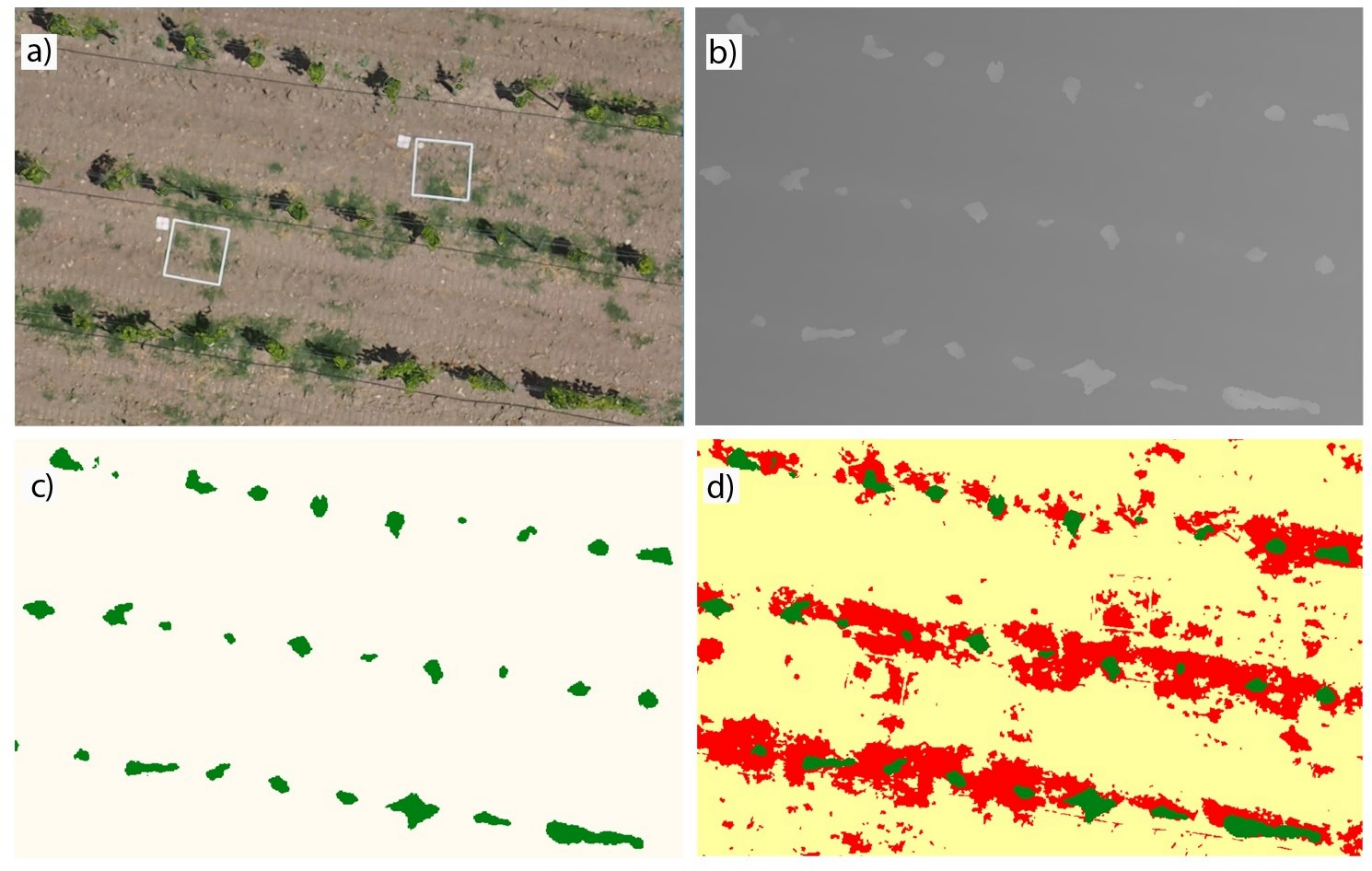
Several stages of the OBIA algorithm for an enlarged view belonging to field A-2016 and RGB camera. **a**) the RGB bands, b) the DSM of the orthomosaic, c) vine line classification (grapevines in green color and no-vineyard objects in white color), and d) classified map (grapevines in green color, bermudagrass patches in red color, and bare soil in yellow color).

Once the VIs that best separated bare soil and bermudagrass were selected, a novel OBIA algorithm was developed to classify the grapevines, bare soil, and bermudagrass using Cognition Network programming language with the eCognition Developer 9.2 software (Trimble GeoSpatial, Munich, Germany). The algorithm is fully automatic and requires no user intervention. Besides this, the same algorithm was used to analyze the orthomosaics generated by each camera, with the only difference being the VI implemented by selecting the optimal one for each. The sequence of phases that compose this algorithm is detailed below:

- *Vine classification*: Height information contained in the DSM model was used to detect and classify grapevine objects (Fig 5B), as fully described in a previous work [3], which first consisted of orthomosaic-image segmentation based on spatial information for object generation (chessboard segmentation). Then, the DSM standard deviation was used to create "vine candidates" that were analyzed at the pixel level to achieve a more refined grapevine classification. Finally, the algorithm classified every pixel as vineyard or not-vineyard by comparing their height value from the DSM with that of the adjacent bare soil. Therefore, spatial information proved to be very suitable for grapevine classification, avoiding errors related to field slope by considering average soil altitude as well as avoiding confusion due to spectral similarities. Finally, the objects in the images were classified as vineyard or not-vineyard objects (Fig 5C).
- *Bermudagrass and bare soil classification*: Once the grapevines were correctly classified, the orthomosaic was segmented using a multiresolution segmentation where the layers Red, Green, and Blue for the RGB camera, and Red, Green, and NIR for the RGNIR camera were weighted to 1, whereas the DSM layer was weighted to 0 in both cases. Multiresolution segmentation is a bottom-up segmentation algorithm based on a pairwise region merging technique in which, based on several parameters defined by the operator (scale, color/shape, smoothness/compactness), the image is subdivided into homogeneous objects. The scale parameter established was 5, whereas 0.3 and 0.5 were chosen for shape and compactness, respectively. These values were chosen after performing several tests for showing a better visual adjustment by delineating bermudagrass patches and bare soil. Therefore, these values could also be used in other vineyards with similar characteristics where bermudagrass classification is required.

Subsequently, the no-vineyard objects, consisting of bare soil and bermudagrass were classified using the VI selected for each camera in the previous section. The optimum ratio value was conducted using an automatic and iterative threshold approach following the Otsu method [60] implemented in eCognition, in accordance with [61]. Finally, a classified map was generated where bermudagrass patches, bare soil, and grapevine objects were defined (Fig 5D).

iii *Site-specific bermudagrass management maps*: After vineyard–weed–bare soil classification, information relative to bermudagrass patches was available such as number, location (X and Y UTM coordinates), and area covered by weed patches and vines from the classified map. As an additional phase of the process, the algorithm has the option to design site-specific bermudagrass management maps that are user-configurable depending on the management strategy. For this purpose, the algorithm created a new level by copying the classified object level to an upper level and a chessboard segmentation was applied to build a user-adjustable grid framework following the grapevine row orientation. In this experiment, a customizable 1 x 0.5 m grid size was selected according to the specifications of the intra- and inter-row weeder usually used in organic vineyards [62]. A hierarchical structure was generated in the inter-row area between the grid super-objects (upper level) and the bermudagrass and bare-soil sub-objects (lower level). Next, the weed coverage (% of bermudagrass) was automatically calculated from the ratio of bermudagrass coverage to total area per grid, as it is considered as one of the main variables in the weed control decision-making process [25]. Thus, based on the information related to weed-free zones and weed-infested zones, site-specific treatment maps were created.

#### Bermudagrass map validation

The accuracy of the algorithm was assessed by comparing the GT data corresponding to bermudagrass infestation and bare soil (manual weed coverage and bare soil area) with the output of every image classification process (estimated bare soil and weed coverage) through a confusion matrix. As commented before, 75% of the GT full datasets corresponding to field A for both 2016 and 2017 were used to assess the classification accuracy. In the case of field B-2017, the GT full dataset was used, so this set of examples was used only to assess the performance (i.e., generalization) of the developed algorithm. The confusion matrix provided overall accuracy (OA) (Eq 2) of each orthomosaic classification, which represented the percentage of correctly classified area (bare soil and bermudagrass); and the producer´s accuracy (PA) that indicated the probability that a classified object actually represents that category, i.e., the category of the ground truth data [63]. The omission error, i.e., the complementary value to PA, was also calculated from the confusion matrix and quantified the proportion of bermudagrass coverage misclassified as bare soil.

$$Overall\;classification\;accuracy\;(\%)=100\times \frac{Area\;correctly\;classified}{Total\;area\;classified}$$ (2)

The methodology to identify vine rows based on DSM information has been validated in a previous study [3], where a high level of precision was reached.

## Results and discussion

### Spectral analysis: Vegetation index selected

Spectral information from every orthomosaic was evaluated to select the VI that best discriminated between the bermudagrass and bare soil, as affected by the spectral range of each camera, i.e., RGB and RGNIR. Significant differences between both classes were observed in all the VI calculated. These results confirmed the potential of discriminating bermudagrass from bare soil by using UAV-images taken at the vegetative stage with any of the cameras (RGB and RGNIR) onboard the UAV, when bermudagrass plants showed a very different green color from the brown of the bare soil. The best results obtained with the M-statistic for images taken with each type of camera were ranked and are shown in Table 2.

**Table 2:** Vegetation indices analyzed with the highest values of M-statistical obtained for each camera.

Camera	Vegetation Index	M-statistical value
RGB	**Excess Green-Red (ExGR)**	**3.50**
	Indices combination1 (COMB1)	3.48
	Excess Red (ExR)	3.16
	Color index of vegetation (CIVE)	3.06
	Excess Green (ExG)	2.87
RGNIR	**Green normalized difference vegetation index (GNDVI)**	**2.27**
	Difference vegetation index (DVI)	2.15
	Chlorophyll index Green (CI)	2.14
	NIR/G	2.14
	NIR-G	2.10

Letters in bold correspond the spectral vegetation indices that showed the highest M values and were then used in the further OBIA algorithm.

According to [64], two classes exhibit moderate separability when M exceeds 1 and good discrimination when it exceeds 2. In this experiment, most of the VIs extracted for each camera achieved M values larger than 2, therefore showing high discriminatory power to separate bermudagrass from bare soil. ExGR showed the best spectral separability in the analysis of the RGB-range, reaching an M value of 3.50, whereas GNDVI was the selected index for the RGNIR-range spectral analysis, as obtained by the highest M value (2.27). As a result of the spectral analysis, ExGR and GNDVI were the optimum VIs selected to carry out the discrimination between both classes for the RGB- and RGNIR-orthomosaic, respectively, thus, the corresponding index was implemented in the classification algorithm developed.

ExGR is a combination of redness (ExR) and greenness (ExG) indices widely used for vegetation identification with visible spectral-index based methods under the assumption that plants display a high degree of greenness due to chlorophyll in the leaves. In this context, [39,65] used ExGR to separate the plants from the soil and residue background in the RGB images. On the other hand, GNDVI has been used to measure several plant parameters including N status [66,67], plant biomass [68], and early disease detection [31] due to the high sensitivity to the chlorophyll concentration variation of this vegetation index. Thus, based on that premise and the results obtained in the spectral analysis in this investigation, GNDVI showed high robustness in the ability of separating bare soil (no chlorophyll) from bermudagrass at the vegetative stage (in green color due to the high concentration of chlorophyll pigment) as a part of the developed algorithm for the image analysis. These results showed the importance of the timing for this analysis, as it is feasible when BG plants are at vegetation phenological stage and green. On the other hand, these VIs would not be suitable for bermudagrass discrimination in another season, e.g. in winter when bermudagrass is dormant (light brown) and shows a similar spectral response to bare soil. At that time, it would therefore be necessary to apply a different analysis such as this one based on texture characteristics [69].

### Image analysis

#### Classified maps

**Fig 6:**
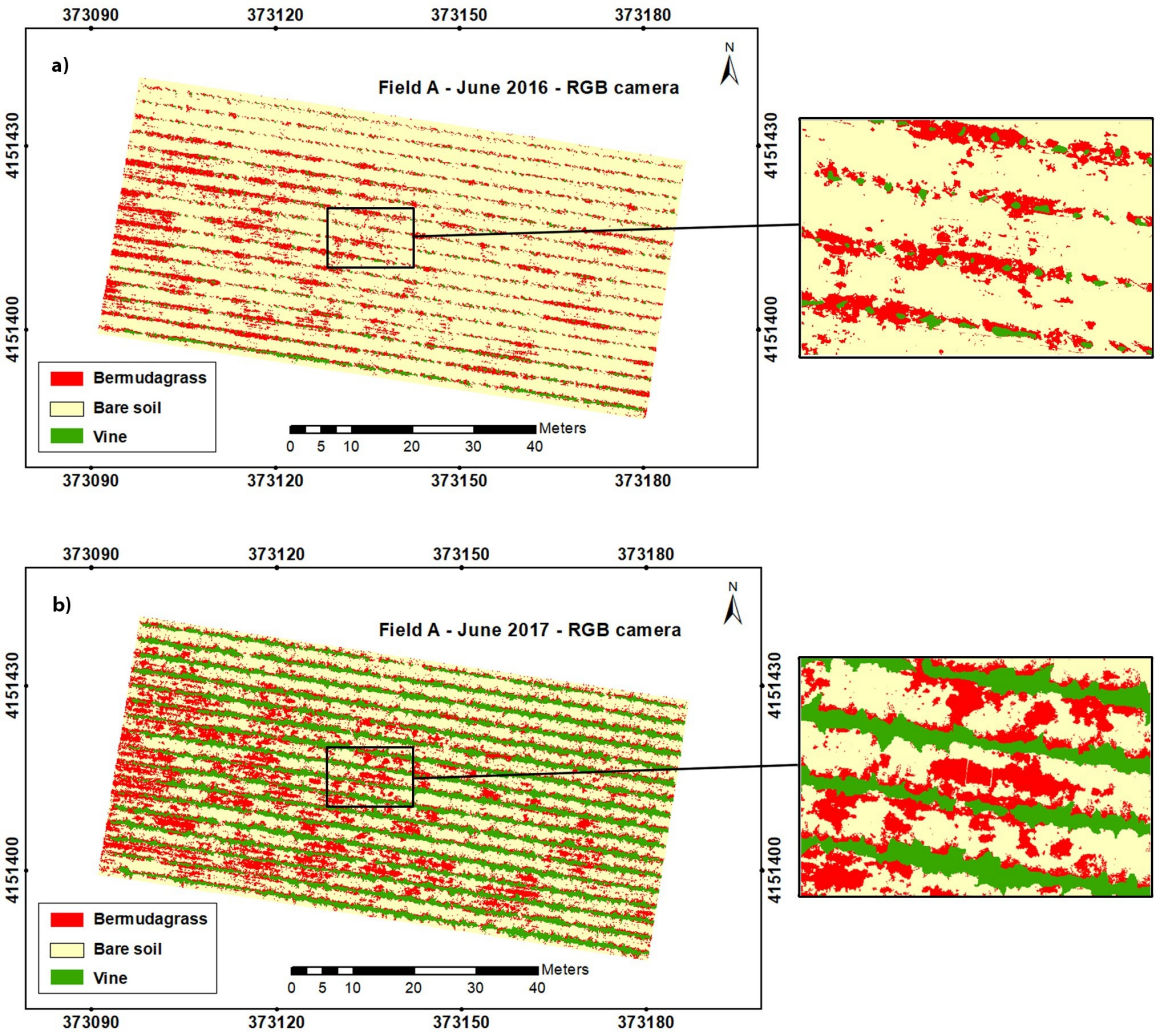
Classified maps developed by the OBIA-algorithm using RGB-imagery for field A in: a) 2016 and b) 2017.

**Fig 7:**
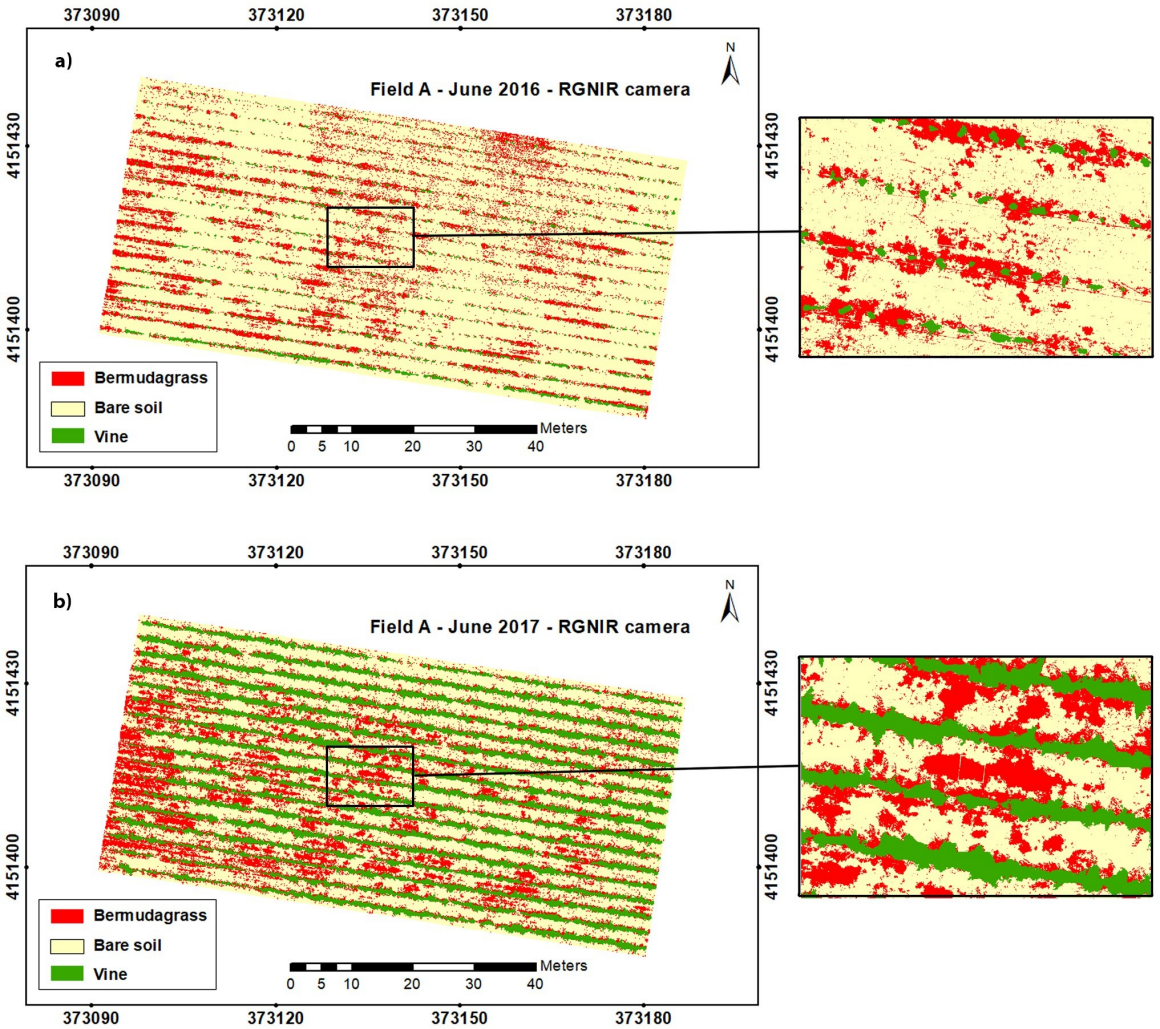
Classified maps developed by the OBIA-algorithm using RGNIR-imagery for field A in: a) 2016 and b) 2017.

After the spectral analysis was carried out, the study focused on image analysis. An OBIA algorithm was developed to parse the orthomosaics as affected by kind of sensor for the suitable discrimination of bermudagrass patches. Next, the algorithm automatically mapped grapevines, bermudagrass, and bare soil by classifying every image object according to these three classes. Thus, a classified map for each field, year, and camera was created (Figs 6 and 7), where clear differences in grapevine size were observed when analyzing the two years studied. Moreover, the developed methodology was able to map bermudagrass within the grapevine rows in the first study year (2016) since at that time the vines were at an initial stage of growth so that the canopy was not closed and it was possible to get information down to ground level. Nevertheless, the growth of the grapevines in the second year (2017) made it unfeasible to obtain that information up to ground level within the row as the vines showed overlapping crowns. Consequently, using the developed UAV-based OBIA algorithm at the proper timing of grapevine growth would enable accurate mapping of weeds within vine rows. The total area occupied by grapevines and bare soil as well as the area infested by bermudagrass was quantified and extracted from these classified maps (Table 3).

**Table 3:** Classified area of grapevine, bermudagrass and bare soil obtained from the RGB and RGNIR images analyses at every location and year studied.

Camera	Field	Year	Classified Area (%)^a^		
			*Vine*	*Bermudagrass*	*Bare soil*
RGB	A	2016	3.4	13.8	82.8
		2017	24.4	21.3	54.3
	B	2017	20.8	21.9	57.3
RGNIR	A	2016	3.7	14.6	81.7
		2017	24.5	19.7	55.8
	B	2017	21.3	20.5	58.2

^a^Percentage of surface occupied for each class respect to total field area.

Similar results of the classified area were obtained by using any of the sensors, e.g., 24.4% and 24.5% for the vine class in field A-2017 when employing the RGB-sensor and RGNIR-sensor orthomosaic, respectively; and similarly, for bare soil in field A-2016, reporting 82.8% and 81.7% of the classified area, which demonstrated the algorithm robustness.

An increase of approximately 21% in the vineyard was observed in the comparison of 2016 and 2017 orthomosaics for both sensors. These differences in grapevine size were the result of usual growth as a relevant rate of development was experienced by vines in those years [70].

The surface infested by bermudagrass also augmented in the context of that temporal comparison, obtaining an increased value of 7.5% when the RGB imagery was analyzed, despite uniform weed management in the inter-row spaces was carried out, This management consisted of biannual tillage and manual mowing using a brush cutter; no synthetic chemicals were used as both fields were organic. Thus, the increase of bermudagrass coverage could be due to inefficient weed management since perennial weeds established by rhizomes or stolons are considered the most difficult to manage in organic orchards, and in fact, they can become a permanent control target as the removal of aerial parts does not eliminate weeds and portions of stolons or rhizomes may re-grow and colonize new areas [71]. According to [72], among the recommendations for bermudagrass management, mowing should be minimized as stolons can cause weed dispersion. They advised a single deep cultivation (up to six inches), avoiding very moist soils, which brings most shoots to the surface to dry them out, and pointed out that this weed management (tilling and drying) did not eradicate seeds in the soil. In addition, deep cultivation risks damaging the roots, trunks, and arms of the grapevines [19]. Other alternatives for weed control include the use of cover crops such as perennial or annual grasses (*Festuca arundinacea* or *Hordeum vulgare*, respectively) or legumes (*Medicago rugosa*), which compete with the bermudagrass and reduce its infestation [17].

Furthermore, a reduction in the area occupied by bare soil was found using any of the sensors, which was quantified as 28.5% for the RGB-orthomosaic image and 25.9% for the RGNIR-orthomosaic.

#### Bermudagrass mapping accuracy

As mentioned in the OBIA algorithm description, the vine class was first separated from the rest of classes using DSM height information as described in [3], where overall accuracy values higher than 93.6% were achieved in the vine classification. The classification statistics of the bare soil and bermudagrass classes obtained in the confusion matrix (OA and PA) for the orthomosaic corresponding to each sensor, field, and year are shown in Table 4. The matrix indicated an overall accuracy higher than 97.7% in all of the cases studied, well above the minimum accepted value standardized at 85% by [73]. These consistent results proved the suitability of the VIs selected in the previous spectral analysis and demonstrated that the VI-based OBIA algorithm correctly identified and mapped the bermudagrass patches in the inter-rows of the vineyards in both years of the study. Moreover, high degrees of producer’s accuracy with values close to or even 100% were achieved in all the studied cases, which corresponded to null or very low values of omission error.

**Table 4:** Classification statistics obtained in confusion matrix for each year, field and camera.

Year	Field	Camera	Producer´s Accuracy (%)		Overall Accuracy (%)
			Bg^a^	Bs	
2016	A	RGB	98.3	99.9	**98.7**
		RGNIR	95.7	99.9	**97.7**
2017	A	RGB	99.6	100	**99.7**
		RGNIR	99.9	99.9	**99.9**
	B	RGB	99.9	100	**99.9**
		RGNIR	99.9	100	**99.9**

^a^Bg: Bermudagrass; Bs: Bare soil. The algorithm was executed with the selected VI for each camera in the previous section, i.e. ExGR for RGB-orthomosaic and GNDVI for RGNIR-orthomosaic.

Similar classification accuracy was achieved using images from both cameras, proving that it is possible to map bermudagrass at the vegetative stage based on RGB-imagery and RGNIR-imagery taken by UAV. For example, 99.6% and 99.9% of PA were obtained for the bermudagrass class using the RGB and RGNIR cameras in field A-2017, respectively; and moreover, OA values of 98.7% and 97.7% were reached for those respective cameras and field in 2016. Therefore, due to the similar results as well as the handling and cheaper costs of the conventional camera, as a preliminary conclusion of this experiment, we recommend the use of an RGB sensor for bermudagrass mapping at the vegetative stage during early summer in vineyards. Thereby, only results for this camera are shown throughout the rest of the manuscript.

The highly accurate results achieved in the image analysis proved that the combination of UAV imagery and OBIA is a suitable tool to map the usual classes including weeds in vineyards. In this context, [25] used a similar image-based UAV technology to discriminate weeds in maize (*Zea mays* L.) fields in the early season obtaining 86% of OA in the confusion matrix; however, the precision of the OBIA algorithm was evaluated by comparing weed coverage over grid units, not over objects. Consequently, the OA was related to the percentage of frames correctly classified (the number of correct frames as a percentage of the total number of sampling frames) and unsuitable spatial accuracy measures for OBIA were performed. In our research, the shape and location of weeds were evaluated, as first proposed by [74], who obtained a high level of agreement in the comparison between the manual weed classification in herbaceous crops and that automatically performed by the OBIA algorithm; however, no matrix confusion was calculated in that experiment. Furthermore, although a confusion matrix was performed in the previous paper for the 3D characterization of vineyards [3], the matrix evaluated the precision in the grapevine vs the non-grapevine classification (composed by inter-row cover crops and bare soil), so this methodology remained non-validated for weed detection in the inter-row of the vineyards.

The omission errors (OE), as complementary to PAs, are shown in Table 5, where values lower than 0.4 were obtained in 2017 for both fields and 1.7 in 2016 for field A. Thus, only 1.7% of the bermudagrass objects were misclassified as bare soil, whereas less than 0.4% of weed patches were misclassified in the rest of the cases, being far below those obtained by [25], who reported values of 17% for frames at moderate weed coverage, and by [26], who obtained a 12% omission error in the classification of grass (*Bouteloua eriopoda* Torrey) using UAV and OBIA techniques. Moreover, no errors were quantified in the bare soil classification. From an agronomic perspective, a key issue for successful management is to report low OE values as it increases the chance of controlling all of the weed patches and also reduces the risk of allowing weeds to go untreated [75]. Therefore, bermudagrass maps obtained from the automatic VI-based OBIA algorithm can be an accurate and suitable tool for farmers to control this species in vineyards.

**Table 5:** Omission error statistics obtained for each year and field using RGB camera.

Year	Field	Omission error (%)	
		Bg^a^	Bs
2016	A	1.7	0.0
2017	A	0.4	0.0
	B	0.1	0.0

^a^Bg: Bermudagrass; Bs: Bare soil.

#### Site-specific weed management

**Fig 8:**
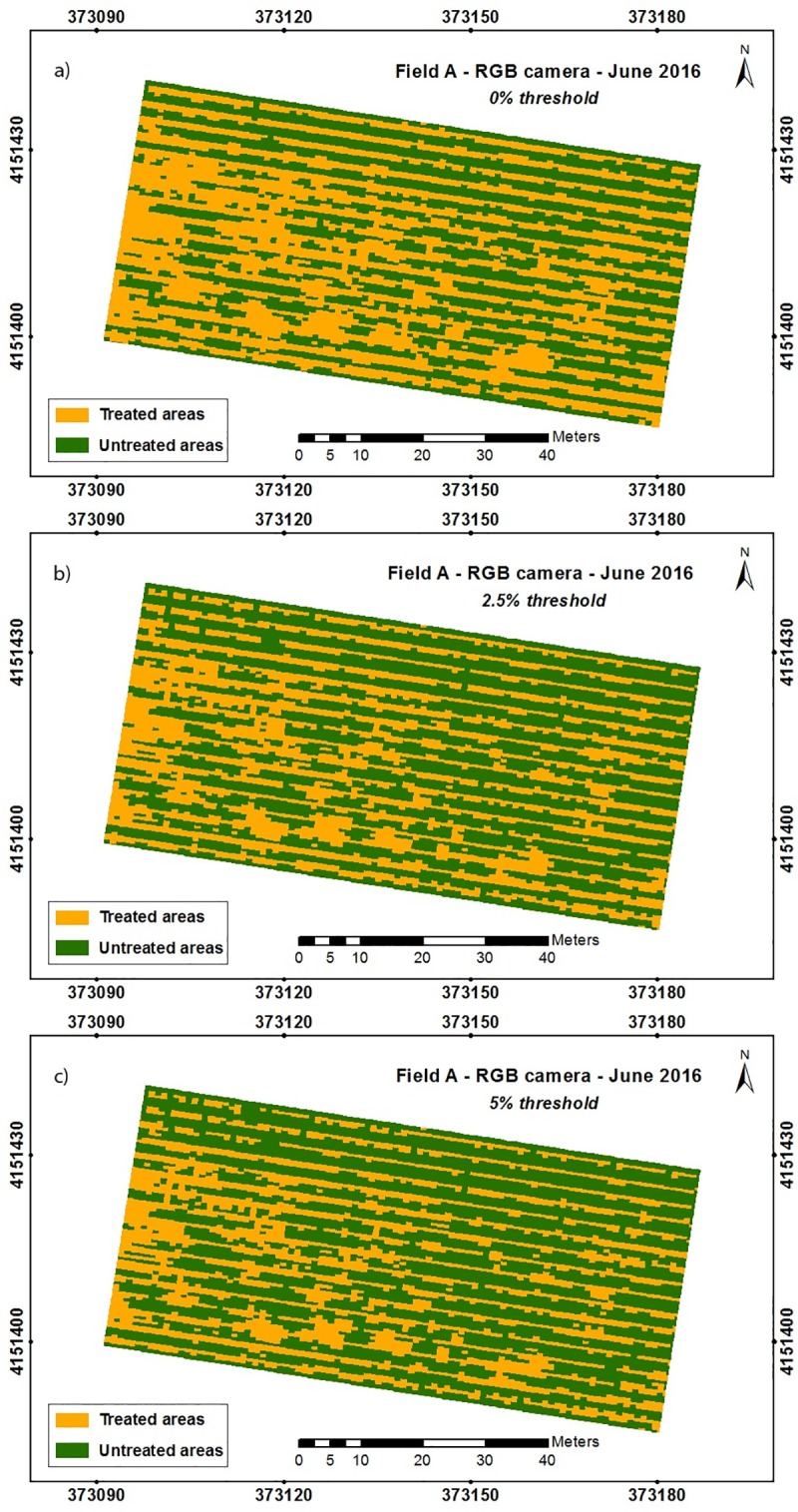
**Site-specific treatment maps for bermudagrass patches in field A-2016 according treatment thresholds: a) 0%, b) 2.5%, and c) 5%.** Only results for RGB camera are shown.

The bermudagrass maps could help farmers improve weed control through a rational-programmed strategy based on site-specific weed management (SSWM), targeting suitable control measures only where they are needed, either intra- or inter-rows. In addition, these maps could also be used to both design a control management strategy for organic vineyards through spraying organic herbicides such as clove oil, acetic and citric acid products [76,77], and using herbicides in the case of non-organic vineyards, according to the weed coverage. In this context, site-specific bermudagrass treatment maps were designed by the OBIA algorithm (Fig 8) based on the weed maps as explained in the Materials and Methods section, through delineating site-specific treatment zones according to the several weed cover thresholds. Three user-adaptable treatment thresholds were selected in this experiment: 0, 2.5, and 5%, where 0% implies that herbicides must be applied in the treatment zone just when there is the presence of bermudagrass, and 5% that the herbicide must be applied when weed coverage is equal or higher than 5%.

The potential herbicide savings, calculated in terms of untreated areas, extracted from the SSWM maps are shown in Table 6. Since savings percentages were calculated based on bermudagrass coverage, savings values varied for each scenario, e.g., the potential savings for field A-2016 consisted of 48.3% from the more conservative prescription maps, since any grid with the presence of bermudagrass was considered a treatment area, while potential savings of 23.4% would be obtained for field B-2017 under the same conservative circumstance. Furthermore, as expected, higher potential savings were observed for higher treatment thresholds [25]. In that sense, about a 14% raise in potential savings was achieved using a 5% weed threshold when compared to the more conservative one for the three cases analyzed. Consequently, the reduction in the bare soil area resulted from the growth of grapevines and the increase in the area infested by bermudagrass.

**Table 6:** Herbicide saving obtained from herbicide application maps as affected by treatment thresholds for RGB imagery by year and field analyzed.

Year	Field	Herbicide saving by treatment thresholds (%)		
		0	2.5	5
2016	A	48.3	58.5	62.2
2017	A	24.4	33.5	38.7
	B	23.4	31.9	36.5

These values correspond to a 1 x 0.5 m grid cell size.

In summary, the combination of UAV imagery and the VI-OBIA algorithm developed provides automatic and accurate bermudagrass mapping. These weed maps could be used to design site-specific bermudagrass management in organic vineyards as well as to create site-specific prescription maps according to weed coverage for non-organic vineyards. These prescription maps could aid in controlling bermudagrass in several agricultural seasons so that the species could be eradicated. This PV-based approach could lead to herbicide reductions, and also optimize fuel, field operating time and cost [74].

## Conclusions

Based on the high competition caused by bermudagrass infestation in the inter-row of vineyards, the possibility of mapping this weed using UAV-imagery was evaluated to facilitate site-specific weed management in the context of PV. Aerial images of several fields were captured using two sensors (RGB and RGNIR) attached to the UAV that allowed us to obtain ultra-high spatial resolution imagery and operate on demand according to the necessities of the grapevines. First, the spectral data analyses showed significant differences between the bare soil and bermudagrass, then ExGR and GNDVI were the optimum VIs selected to carry out the discrimination between both classes for the RGB- and RGNIR-orthomosaic, respectively. Second, an accurate and fully automatic VI-based OBIA algorithm was developed to map bermudagrass infesting the inter-row of vineyards, where the optimum VI for each camera was implemented. Grapevines were mapped using photogrammetric-based DSMs, thus avoiding misclassification due to the spectral similarity between the vines and bermudagrass. High values of map classification accuracy (>97.7%) were achieved with each of the cameras, proving that it is possible to map bare soil, grapevines, and bermudagrass at the vegetative stage based on RGB- and RGNIR-imagery. Thus, due to the similar results and handling and cheaper cost of the conventional camera, the use of an RGB sensor was recommended for that objective.

The analysis of the classified area from maps allowed us to quantify grapevine growth in those years and revealed the area infested by bermudagrass. Thus, these bermudagrass maps generated by the VIs-based OBIA algorithm could help farmers improve weed control in organic vineyards through a well-programmed strategy based on site-specific weed management (SSWM). Moreover, site-specific bermudagrass treatment maps, according to the weed coverage of the field, were designed by the algorithm to spray herbicides to be used for non-organic vineyards in the context of precision viticulture. Using these prescription maps could aid in controlling bermudagrass across several agricultural seasons and eradicating this species.

This PV-based approach could reduce herbicide use, and optimize fuel, field operating time, and costs.

## Supporting information

S1 Table Spectral values for every digitized pixel of bermudagrass and bare soil obtained with RGB camera in 2016. (XLSX)

S2 Table Spectral values for every digitized pixel of bermudagrass and bare soil obtained with RGNIR camera in 2016. (XLSX)
